# The EyeFlowCell: Development of a 3D-Printed Dissolution Test Setup for Intravitreal Dosage Forms

**DOI:** 10.3390/pharmaceutics13091394

**Published:** 2021-09-03

**Authors:** Tobias Auel, Linus Großmann, Lukas Schulig, Werner Weitschies, Anne Seidlitz

**Affiliations:** 1 Center of Drug Absorption and Transport, Department of Biopharmaceutics and Pharmaceutical Technology, Institute of Pharmacy, University of Greifswald, 17489 Greifswald, Germany; tobias.auel@uni-greifswald.de (T.A.); linus.grossmann@uni-greifswald.de (L.G.); werner.weitschies@uni-greifswald.de (W.W.); 2 Department of Pharmaceutical and Medicinal Chemistry, Institute of Pharmacy, University of Greifswald, 17489 Greifswald, Germany; lukas.schulig@uni-greifswald.de

**Keywords:** in vitro model, in vitro drug release, intravitreal implants, SLA 3D-printing, triamcinolone acetonide, USP apparatus 4, USP apparatus 7, vitreous substitute, dissolution

## Abstract

An in vitro dissolution model, the so-called EyeFlowCell (EFC), was developed to test intravitreal dosage forms, simulating parameters such as the gel-like consistency of the vitreous body. The developed model consists of a stereolithography 3D-printed flow-through cell with a polyacrylamide (PAA) gel as its core. This gel needed to be coated with an agarose sheath because of its low viscosity. Drug release from hydroxypropyl methylcellulose-based implants containing either triamcinolone acetonide or fluorescein sodium was studied in the EFC using a schematic eye movement by the EyeMovementSystem (EyeMoS). For comparison, studies were performed in USP apparatus 4 and USP apparatus 7. Significantly slower drug release was observed in the PAA gel for both model drugs compared with the compendial methods. Drug release from fluorescein sodium-containing model implants was completed after 40 min in USP apparatus 4, whereas drug release in the gel-based EFC lasted 72 h. Drug release from triamcinolone acetonide-containing model implants was completed after 35 min in USP apparatus 4 and after 150 min in USP apparatus 7, whereas this was delayed until 96 h in the EFC. These results suggest that compendial release methods may overestimate the drug release rate in the human vitreous body. Using a gel-based in vitro release system such as the EFC may better predict drug release.

## 1. Introduction

The incidence of eye diseases has increased steadily in recent years as a result of demographic change. In particular, diseases of the posterior segment of the eye, such as diabetic retinopathy, macular edema, or age-related macular degeneration, are more and more common [1,2,3]. Intravitreal therapy as a minimally invasive procedure, in which suspensions, solutions, or implants containing antibodies like bevacizumab (Avastin^®^) or glucocorticoids like dexamethasone (Ozurdex^®^) or triamcinolone acetonide (TA) are injected into the human vitreous body, is becoming increasingly crucial for the treatment of such clinical conditions [4]. Implants, in particular, provide better patient compliance because of their sustained release, as the intervals between the individual applications may be extended [5].

For ethical and practical reasons, in vivo studies in humans in preclinical development of new intravitreal dosage forms are complicated. On the one hand, an in vivo determination of the drug in the vitreous body is almost impossible. On the other hand, the human eye is a susceptible organ, where even minor damages can lead to visual impairment. Therefore, the use of animal models plays a significant role in preclinical development. Aside from rats and mini-pigs, rabbits are the most commonly used animal species [6]. Even if animal models provide preliminary conclusions regarding the behavior of dosage forms in the human vitreous, it must be taken into account that the physiological conditions differ to a variable extent; that is, different vitreous volumes, aqueous flows, and vitreous diffusional pathlength between the species may complicate the transferability of results from in vivo studies [7,8]. Another important aspect is the age-related liquefaction of the human vitreous body. Whereas this frequently occurs in humans with increasing age, possibly leading to a different release and distribution behavior of dosage forms, young animals used in in vivo studies have a more gel-like vitreous body that cannot reflect this fact [9,10,11]. For this reason, the combination of in vivo animal studies with more biorelevant in vitro models is a possible option to reduce the number of animal studies and obtain a better first idea of the behavior of new drug formulations in the human vitreous body.

Compendial release apparatuses of the United States Pharmacopeia (USP) such as the flow-through cell (USP apparatus 4) or the reciprocating holder (USP apparatus 7) are the first approach for dissolution testing of new intravitreal dosage forms. Both are recommended, for example, for in vitro release studies of implants [12]. The so-called shake-flask method, in which implants are incubated with release medium in tubes under agitation, is also commonly found in the literature [13,14]. With these methods, it is possible to represent lower liquid volumes, as they are present at many application sites of implants. Nevertheless, when it comes to simulating the physiological conditions of the human vitreous body, unique characteristics such as the gel-like consistency or the aqueous flow are neglected here. There are only a few in vitro models that try to simulate the behavior of drug formulations in the human vitreous. One of them is the PK-Eye developed by Awwad et al., which attempts to simulate the clearance of drugs via the aqueous flow-through the anterior chamber of the eye [15]. Another model is the EyeMovementSystem (EyeMoS) created by Loch et al. [16] and adjusted by Stein et al. [17], which takes the influence of simulated eye movements on the distribution and release of drugs into account. Both models investigate the influence of individual aspects on the distribution behavior of active ingredients in a simulated human vitreous body. However, they are nevertheless fairly limited regarding the information they provide about the release of the drugs from different dosage forms.

This work aimed to develop a gel-based flow-through system and combine it with the previously developed EyeMoS so that continuous dissolution testing is possible over a more extended time. Until now, it was necessary for dissolution studies in the EyeMoS either to perform multiple studies with different endpoints or to transfer the test objects into fresh gels at defined time points. This transfer meant that the dosage form was subjected to mechanical stress. By adding a flow-through system around the gel, sampling from an external vessel should be possible without affecting the dosage form.

For this purpose, model implants based on hydroxypropyl methylcellulose (HPMC) containing fluorescein sodium (FS) or TA were manufactured by hot-melt extrusion. In the first studies, the model was tested with the analytically accessible, hydrophilic model substance FS, which is used as a dye in fluorescein angiography for staining tissue layers, among other applications [18]. The glucocorticoid TA is used as a vascular endothelial growth factor (VEGF) inhibitor in the therapy of diseases of the posterior segment of the eye [19].

The drug release from the implants was investigated in the newly developed so-called EyeFlowCell (EFC) after injection into a gel-like core. For this purpose, a polyacrylamide (PAA) gel developed by Loch et al. was used, corresponding to the human vitreous body in essential characteristics such as water content, pH, density, and viscosity [16]. As the PAA gel used by Loch et al. has a low viscosity, a setup had to be found to prevent it from being washed away by the flowing media. For this purpose, the PAA gel was coated with a form-stable agarose sheath and the dual gel was placed centrally in a 3D-printed basket so that the release medium could flow around it. By combining it with the EyeMoS, the influence of schematic eye movement on drug release was investigated. Moreover, for reasons of comparison, dissolution studies were performed in the USP standard apparatuses 4 (flow-through cell) and 7 (reciprocating holder).

## 2. Materials and Methods

### 2.1. Materials

Sodium chloride, potassium chloride, disodium hydrogen phosphate, and potassium dihydrogen phosphate as components of phosphate buffered saline (PBS) pH 7.4 were purchased from AppliChem (Darmstadt, Germany). A PAA gel developed by Loch et al. [16] simulating the vitreous body was prepared according to the composition given in Table 1. Rotiphoresis gel 30 (37.5:1), ammonium peroxodisulfate, and tetramethylethylenediamine were purchased from Carl Roth (Karlsruhe, Germany). Agarose for the PAA gel-sheath was purchased from Sigma Aldrich, Germany. Model implants were prepared via hot-melt extrusion of FS (Sigma Aldrich, St. Louis, MO, USA) or TA (Caelo, Hilden, Germany) as active pharmaceutical ingredients, hydroxypropyl methylcellulose (HPMC; Affinisol 100LV/Affinisol 15LV; Dow Chemicals, Midland, TX, USA) and polyethylene glycol (PEG) 6000 (Carl Roth). All chemicals and solvents for the high-performance liquid chromatography (HPLC) were of analytical quality. A standard Formlabs Clear Resin (Formlabs, Somerville, MA, USA) was used for the 3D-printed EFC.

### 2.2. Methods

#### 2.2.1. Preparation of the Model Implants

For the preparation of the drug-loaded model implants, the components listed in Table 2 were mixed, dried in an oven for 24 h at 40 °C, and then extruded with a twin-screw extruder (Three-Tec ZE12, Seon, Switzerland) through a 0.5 mm nozzle. The powder inlet was cooled to 15 °C, and the barrel was heated to 180 °C. A Three-Tec conveyor belt was used to stretch the extrudates. The produced filaments were cut to a length of approximately 6 mm with a disposable scalpel, and their mass was determined individually using a Sartorius SE2 ultra-micro balance (Sartorius, Goettingen, Germany).

#### 2.2.2. Fabrication of the EyeFlowCell

**Figure 1:**
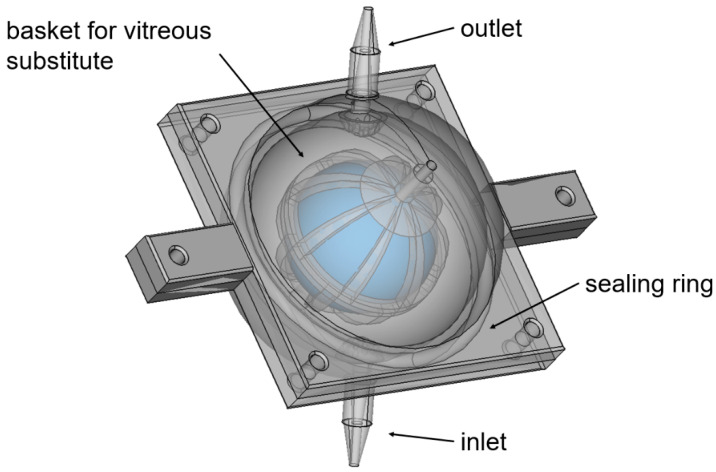
Schematic view of the EyeFlowCell. The vitreous substitute should be placed centrally in the basket allowing the flow to pass through the inlet to the outlet and surround the substitute.

The 3D-printed dissolution chambers, the so-called EyeFlowCell, were designed with FreeCAD (version 0.17), sliced with Preform (version 3.4.5) software, and printed with a Formlabs Form 3 (Formlabs, Somerville, MA, USA) stereolithography printer. A clear resin was used as material for all cells. A schematic view of the intended design is shown in Figure 1. The chamber was developed to consist of a closed bottom side and a top side with an injection channel for injecting the dosage forms to be tested and a basket keeps the vitreous substitute centered in the middle. Sealing is achieved by a self-manufactured sealing ring made of silicone; the injection channel is closed by a plug so no medium can leak out this way. In contrast to conventional flow-through cells, the dissolution chamber is perfused with a release medium from the bottom inlet to the top outlet. Special holders were printed from polylactic acid (Formfutura, Nijmegen, The Netherlands) using an Ultimaker 3 Extended (Ultimaker, Ultrecht, The Netherlands) to mount the EFC on the EyeMoS.

#### 2.2.3. Fabrication of the Vitreous Substitute

**Figure 2:**
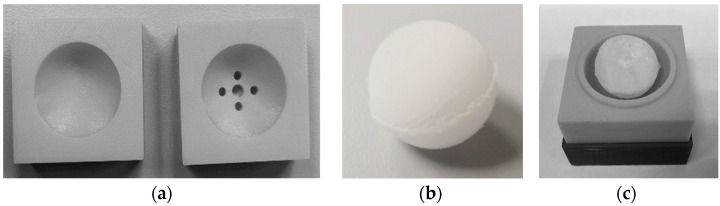
(**a**) Silicone molds for the production of the polyacrylamide (PAA) gel vitreous body substitutes; (**b**) frozen PAA gel core; (**c**) mounted PAA gel-core before coating with agarose-solution.

In order to prevent the PAA gel from washing out, a method to produce a sheath around it had to be developed. To mimic the volume of the human vitreous body, a spheric 4 mL gel body of the PAA gel was cast in silicone molds (r = 10 mm; Figure 2a) and frozen at −80 °C for at least 2 h (Figure 2b). A 2% agarose solution in PBS 7.4 mixture was heated on a heating plate to 100 °C under agitation until the agarose was completely dissolved and then cooled down to 50–60 °C. Evaporation loss was corrected and the frozen PAA gel was fixed centrally with a metal rod in a larger silicone mold (r = 14 mm; Figure 2c). This mold was used to coat the PAA gel with the hot agarose solution and create a uniform, 4 mm thick sheath. The dual gel was stored at 8 °C for at least 2 h.

#### 2.2.4. In Vitro Drug Release Studies Using Compendial Methods

In vitro release testing of the FS and TA-containing HPMC implants in USP apparatus 4 was performed using flow-through cells under sink conditions at room temperature in a closed system. A magnetic stirrer stirred the test volume of 100 mL PBS in a vessel at 250 rpm. An Ismatec IPC peristaltic pump was used to circulate the release medium at a flow rate of 5 mL/min through the cells. Samples of 1 mL were withdrawn at predetermined time points from the vessel and replaced with fresh PBS after sampling. In vitro release of TA-containing HPMC implants was furthermore studied under sink conditions in a USP 7 apparatus (400-DS Dissolution Apparatus 7, Agilent Technologies, Santa Clara, CA, USA) at room temperature. Cells were filled with a volume of 10 mL of PBS and each cell was loaded with a 12-port mesh basket dipping at 30 dips per minute through an externally controlled magnetic plate. A sample volume of 1 mL was withdrawn at each time point, and the remaining volume was discarded and replaced with 10 mL of fresh PBS.

#### 2.2.5. In Vitro Drug Release Studies in the EyeFlowCell

**Figure 3:**
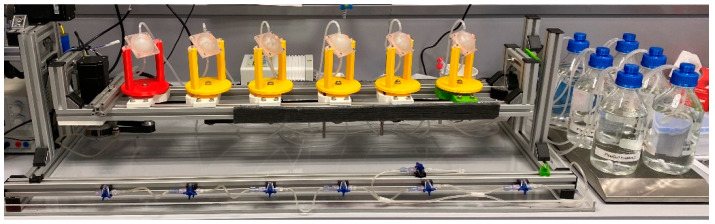
Setup of the EyeFlowCell combined with the EyeMoS.

The release testing of the drug-loaded model implants was performed in the newly developed system combined with or without the EyeMovementSystem (EyeMoS). For both methods, 100 mL PBS 7.4 was used as the release medium. The medium container was stirred at room temperature by a magnetic stirrer at 250 rpm. An Ismatec IPC peristaltic pump was used to circulate the release medium at a flow rate of 5 mL/min through the chambers. A picture of this setup is shown in Figure 3.

For the release in the EFC, the gel vitreous bodies were prepared as described above and inserted into the dissolution chambers. The drug-loaded implants were injected into the gel vitreous bodies with a Sterican cannula (0.5 × 16 mm) through the injection channel. The cannula was fitted with a spacer to ensure reproducible injection, placing the implant centrally in the vitreous substitute. For dissolution testing with the simulated eye movement, the repetitive 24 h scheme given in Table 3 was used, which was also used in previous works [16,17]. To determine the residual drug content in the gel, TA or FS was extracted from the gel with 10 mL of acetone. Extraction was carried out in an incubator at room temperature for 24 h and 150 rpm. The PAA gel collapsed upon exposure to the solvent and was removed with the parts of the agarose sheath. The acetone was then evaporated, and the residue was taken up in 10 mL PBS and measured by high-performance liquid chromatography (TA) or fluorescence spectroscopy (FS).

#### 2.2.6. Quantification

TA was quantified using a Shimadzu Nexera XR Modular high-pressure liquid chromatography system (Shimadzu, Kyoto, Japan) consisting of a SIL-20ACxr autosampler, a CTO-10AC column oven, SPD-M20A diode array detector, DGU-20A3R degasser, CBM-20A system controller, and LC-20AD pumps. A Kinetix Polar C18 column (2.6 µm, 150 × 2.1 mm; Phenomenex, Torrance, CA, USA) was used. The column oven was heated to 40 °C, and the wavelength of the detector was set to 238 nm. The mobile phase consisted of 30% millipore water and 70% methanol, both mixed with 0.5% formic acid. The injection volume was 20 µL, the flow rate was 0.45 mL/min, and the retention time was approximately 1.8 min. The concentration range for the calibration was 0.1–10 µg/mL with a coefficient of determination (R^2^) of 0.999. The evaluation was performed with the software LabSolution (Shimadzu).

FS was determined by UV/vis spectrometry using a Cary 60 spectrophotometer (Agilent, San Diego, CA, USA) with a fiber optic-based system (slit width 10 mm). In a two-minute interval, measurements were carried out at a wavelength of 490 nm (λ_max_ of fluorescein) and 600 nm (baseline correction). The calibration range was 0.1–10 µg/mL (coefficient of determination R^2^ = 0.999). All FS experiments were performed under protection from light.

## 3. Results

### 3.1. Preparation of the Vitreous Substitute

**Figure 4:**
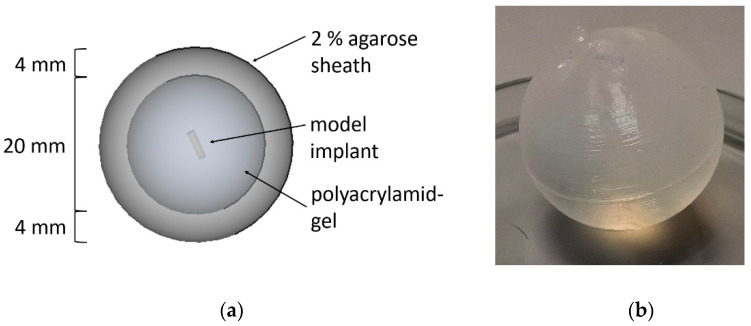
(**a**) Schematic view of the dual gel: the inner polyacrylamide gel core simulates the human vitreous body. Dosage forms like implants or suspensions can be injected into the polyacrylamide gel. The core is coated with a 4 mm thick 2% agarose sheath to maintain the form. (**b**) Photography of the agarose-coated polyacrylamide gel.

The PAA gel used to simulate the vitreous body had to be covered with a form-giving agarose sheath because of its low viscosity. For this purpose, the frozen PAA gel core was coated with a warm agarose solution in a special silicone mold. On contact with the gel, this solution cooled and formed a 4 mm thick agarose sheath. An agarose solution of 2% proved to be practicable in handling. Cracks occurred in the sheath at lower concentrations, so washing out of the gel core could not be avoided entirely. At higher concentrations, the warm agarose solution was too viscous to cast a uniformly thick sheath without defects. Figure 4a shows a schematic view of the dual gel. The coated dual gel is shown in Figure 4b.

**Figure 5:**
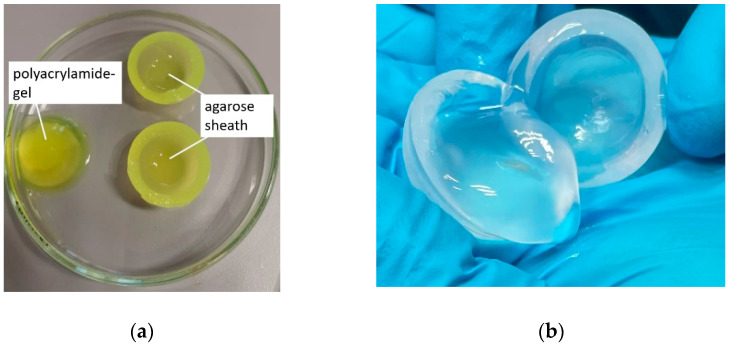
(**a**) Cut open agarose sheath after the end of a fluorescein sodium dissolution study; (**b**) cut open agarose sheath after the end of a triamcinolone acetonide dissolution study.

Even after the release studies, both the inner PAA gel and the outer agarose sheath were intact. Images of vitreous substitutes that were cut in half after a release experiment are shown in Figure 5. There is a clear separation between the viscous PAA gel and the form-stable agarose sheath.

### 3.2. Fabrication of the EyeFlowCell

**Figure 6:**
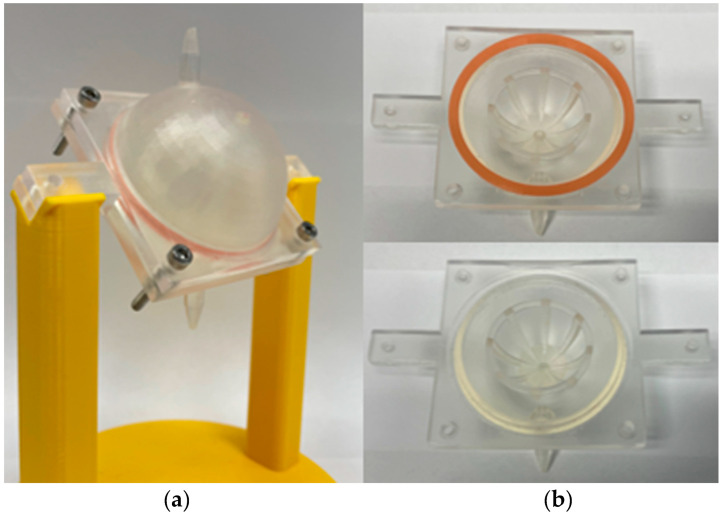
3D-printed EyeFlowCell; (**a**) complete cell mounted on a 3D-printed holder; (**b**) separate top and bottom side of the EyeFlowCell.

The 3D-printed dissolution chamber, called EyeFlowCell, is depicted in Figure 6a. Printing was achieved in a suitable resolution and the planned properties were achieved. The dimensions of the EFC are 60 mm × 60 mm × 52 mm (width, length, height). The chamber volume is 51 mL, of which 11.5 mL is occupied by the inner basket for the vitreous substitute. The central basket for holding the vitreous substitute has an inner radius of 14 mm. The basket itself consists of eight bars with a width of 2.8 mm, which are connected at the top in a 1.4 mm thick ring. Leak tightness was achieved with the custom-made sealing ring. The upper side of the model as shown in Figure 6b has an injection channel with a diameter of 3.5 mm, intended for direct injection of the dosage form to be tested into the inner vitreous substitute. The cell is attached to a holder via two cantilevers so that the orientation allows a vertically upward flow through the cell.

### 3.3. Drug Loaded Model Implants

**Figure 7:**
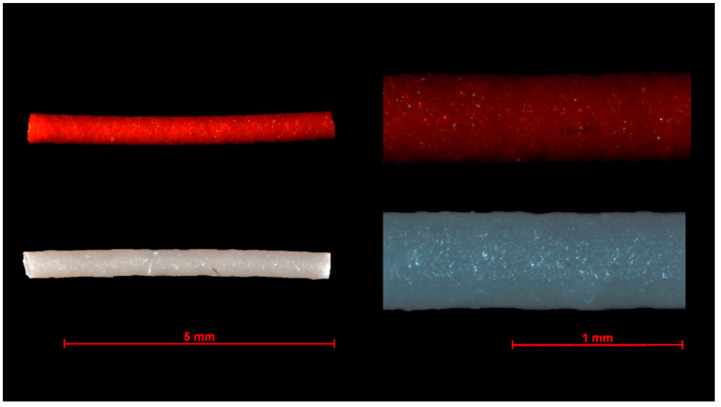
Microscopic images of extruded fluorescein sodium and triamcinolone acetonide HPMC implants.

TA- and FS-loaded implants based on HPMC were successfully extruded. Microscopic images of the extruded implants are shown in Figure 7. The filaments exhibit a relatively uniform diameter of 0.49 mm with a homogeneous matrix and a slightly rough surface. The length of the cut implants was approximately 6 mm. The actual drug loading of the implants was 29.5 ± 1.4% for the implants with FS and 17.3 ± 0.8% for those with TA. In relation to the powder mixture, the drug content of the FS filaments deviates significantly. On the one hand, thermal stress during extrusion and, on the other hand, photoinstabilities could be responsible for this. Before the dissolution experiments, the mass of the investigated implants was determined and used for calculating the drug content. The average mass of FS implants was 1.627 ± 0.103 mg containing 0.480 ± 0.030 mg FS, and the TA implants weighed 1.421 ± 0.219 mg with an amount of 0.246 ± 0.038 mg TA incorporated.

### 3.4. Dissolution Studies

**Figure 8:**
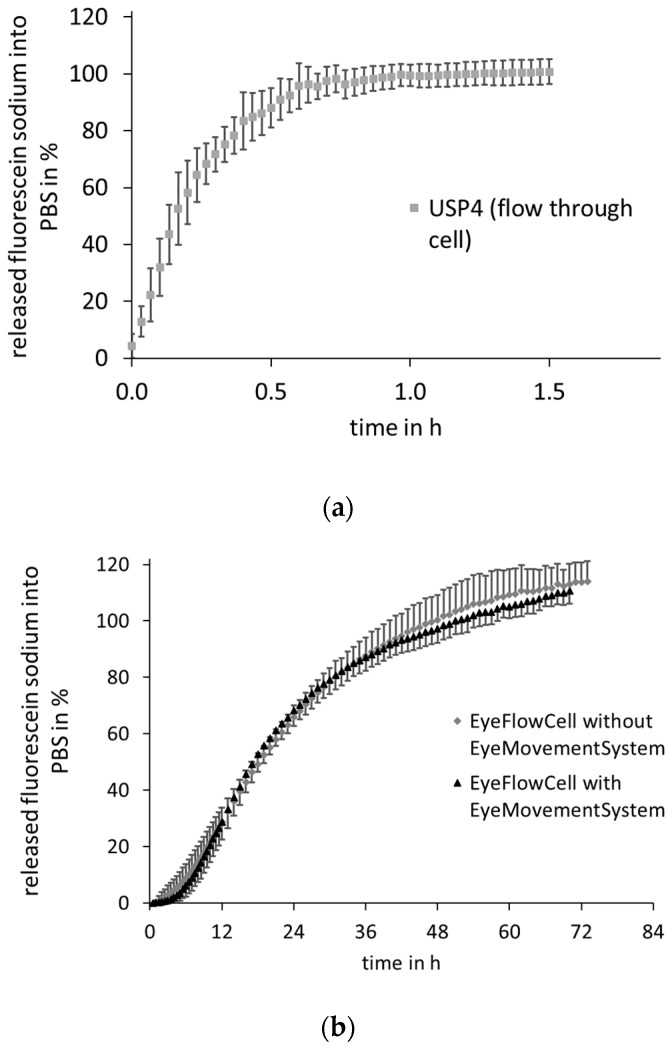
Mean fluorescein sodium release profiles obtained from HPMC model implants in PBS 7.4; (**a**) in the USP apparatus 4 (RT, 250 rpm) *n* = 5 ± SD; (**b**) in the EyeFlowCell with and without agitation with EyeMovement System (RT, 250 rpm) *n* = 5 ± SD.

**Figure 9:**
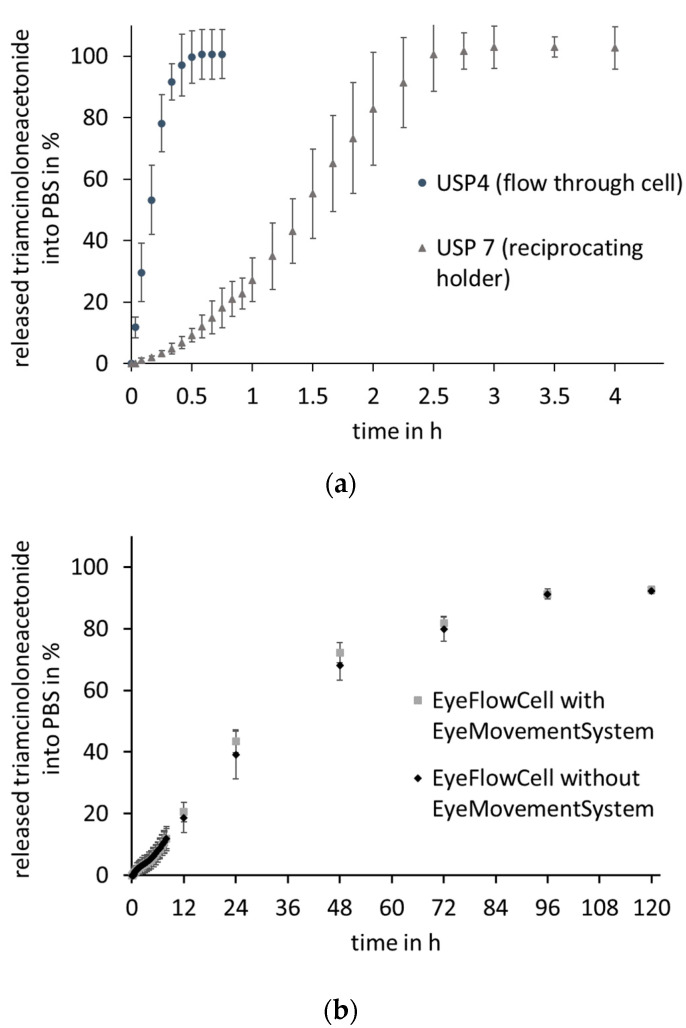
Mean triamcinolone acetonide release profiles obtained from HPMC model implants in PBS 7.4; (**a**) in the USP apparatus 4 (RT, 250 rpm) and USP apparatus 7 (RT, 30 dips/min), *n* = 5 ± SD; (**b**) in the EyeFlowCell with and without agitation with EyeMovementSystem, *n* = 5 ± SD.

The drug release of FS- or TA-containing HPMC implants was tested in the EFC, as shown in Figure 8 (FS) and Figure 9 (TA). For both the hydrophilic FS and the more lipophilic TA, no difference can be seen between the respective profiles in the EFC independent of whether or not the movement was applied. The release of the FS-containing HPMC implants after 72 h was 110.0 ± 4.4% with EyeMoS versus 113.1 ± 7.2% without EyeMoS. In comparison, even minimally less drug is released without the movement by the EyeMovementSystem. However, the slight difference shows that the movement does not seem to have any influence in this experiment. Moreover, there is no difference in the release profiles of the TA-containing HPMC implants. After four days, a plateau was reached for both release profiles in the EFC. At this time, 91.3 ± 1.6% was released when testing with movement, while without movement, 91.3 ± 0.9% of the theoretical drug load was released.

Comparing the profiles with those obtained using compendial methods, a slower release can be seen in the EFC for both drugs. While for the TA-containing implants in the USP apparatus 7, there is a low release at the beginning, in the USP apparatus 4, a rapid increase in the amount released can be seen directly for TA as well as for FS. In USP apparatus 4, the plateau and thus the complete release of TA with 100.5 ± 8.1% is even reached after 35 min, while in USP apparatus 7, the reciprocating holder, 100.6 ± 12.0% is released after 150 min into the surrounding medium. The release from the FS-containing implants reached a plateau of 95.5 ± 4.5% after approximately 40 min in USP apparatus 4.

## 4. Discussion

Monographed pharmacopeia release systems offer many advantages for the in vitro characterization of dosage forms. They assure defined and reproducible conditions for drug release from different dosage forms. As a result, they provide comparability of collected data. However, these systems usually have little in common with the conditions at the respective application sites. Therefore, they offer great advantages for quality control, but lack predictability of the actual behavior of the dosage form in vivo. The general approach for developing a gel-based flow-through cell is based on the assumption that the degradation, swelling, and thus release behavior of dosage forms like implants depends on the medium surrounding them. In the case of intravitreal application, this is the gel-like vitreous body. This site of application is poorly reflected by compendial release systems, leading to differences in in vitro behavior compared with the situation in vivo. Suitable systems for continuous dissolution studies, which provide a better preliminary understanding of how the dissolution might occur in vivo, are lacking so far. While there are several approaches for human vitreous body substitution in vivo, in vitro drug release in these has not been studied in detail so far [20,21]. Apart from the PAA gel by Loch et al. used here, which has already been used for release studies, and another vitreous substitute developed by Awwad et al. from hyaluronic acid and agar, which was used to determine the clearance of PLGA microparticles or antibodies, there is little literature on in vitro experiments. One reason for this could be the problems associated with sampling from gels.

The use of 3D-printing is beneficial for dissolution test setup development. The individual options for design and layout allow new dissolution test models to be adapted quickly and easily, depending on requirements. The stereolithography printing used here enables the precise production of fine models. In previous works, it has been used to produce a Franz cell for permeation studies or to print tablets containing drugs, for example, [22,23]. The EFC is intended to simulate the human vitreous body, which is why a gel was used as a dissolution medium to simulate the human vitreous body, resembling the vitreous body in some essential properties [16]. In the human vitreous body, the gel-like structure is formed by an interaction of primary collagen and hyaluronic acid [24,25]. In contrast, in the PAA gel, this is achieved by crosslinking acrylic amide with tetramethylethylenediamine. The negative charge of the glycosaminoglycan hyaluronic acid is not considered here, for which interactions and inhibition of the diffusion of cations have been described [26]. This point should be an approach for future developments to revise the inner gel core, the in vitro vitreous substitute.

In order to maintain sink conditions, the use of a flow-through system around this gel was chosen. Moreover, this offers the advantage of sampling from a liquid medium, as the determination of the release into gels usually requires extraction for sampling, and thus leads to multiple experiments with different endpoints. Compared with USP apparatus 4, however, the EFC requires a larger minimum volume of the release medium. Owing to the chamber volume of 51 mL and the tubing system, at least 100 mL of release medium is required, whereas in USP apparatus 4, 50 mL or even less is sufficient depending on the cell and tubing design. Because the PAA gel used as the vitreous substitute possesses a low viscosity, it had to be enclosed in a 2% agarose coating to prevent it from being liquefied and washed away by the flow. Preliminary tests have shown that a sheath thickness of 4 mm is necessary to produce a uniform reproducible shell. A spherical coating was chosen because it visually reflects the almost spherical vitreous body and ensures simple and reproducible handling. Preliminary experiments with dye solution (data not shown) have indicated that the diffusion of the drug from the inner PAA gel into the dissolution medium is not significantly hindered.

To test this system, simple model implants were produced from HPMC and FS or TA. HPMC is a water-soluble polymer commonly used in hot-melt extrusion, which has excellent properties for processing. FS was chosen as an analytically well-accessible hydrophilic model substance. The glucocorticoid TA belongs to the VEGF inhibitors and is used as an intravitreal suspension for various diseases like macular edema [27], and was thus selected as a model substance. The length and diameter of the fabricated implants were based on the intravitreal implant Ozurdex^®^ (Allergan), which is approved in Europe.

An extreme discrepancy in dissolution times is noticeable when comparing the dissolution profiles between the EFC and the standard apparatuses. Because of the flow of the medium in USP apparatus 4 and the dipping in USP apparatus 7, the release is completed within 35–150 min, whereas this takes several days in the EFC. This time difference can be attributed to the movement of the dosage form in the medium associated with convective transport of the drug after dissolution, which does not occur when gels are used as injection sites. Another reason for the slower drug release in the EFC could be the dissolution of the implants. As these are immobilized in the gel and degradation products also have to diffuse out of the PAA gel core, slower degradation may result in slower drug release. Moreover, no diffusion of the drugs is required in the compendial setups, so that the released drug can be determined without delay. Comparing the two releases in the EFC, it can be seen that the applied movement had no influence on the release rate in these first investigations. All release profiles in the EFC can be divided into three phases: at the beginning, there is a lag-time of 1.5–2 h, during which almost no drug is released into the medium. In this phase, the drug is most likely distributed in the inner gel core, and is thus not yet detectable in the surrounding medium. Subsequently, the release curves increase constantly over several days until they reach a plateau.

Because diffusion processes play a role in the distribution within the gel and between the different media and, according to Fick’s law, these are only driven by the concentration gradient, this result was expected. These results demonstrate that the PAA gel, intended to simulate the human vitreous body, can significantly slow down drug release from HPMC implants. Assuming that the dosage form behaves approximately the same in the human vitreous, it can be hypothesized that compendial methods significantly overestimate the rate of drug release for both hydrophilic and lipophilic drugs, at least for the release from HPMC implants tested here. However, to confirm this hypothesis and the suitability of EFC to predict the in vivo behavior of dosage forms, in vivo release data are required. Diffusion, as well as convection, plays an essential role in the human vitreous. The elimination of drugs occurs either via the posterior chamber of the eye or the retina, thus the drug has to be distributed in the vitreous body [28]. The distribution processes that take place in the vitreous body could thus be roughly simulated with the distribution in the gel as a vitreous body substitute. Because of the limited volume of the PAA gel, solubility in it could be a limiting factor. Even if sink conditions exist in the release medium, non-sink conditions in the PAA gel could affect the concentration gradient between the vitreous substitute and the dosage form, thus slowing down release. It may be expected that this effect is more pronounced for drugs with low solubility in the water-based gel.

The use of the EFC is not only limited to implants owing to the PAA gel core. An investigation of suspensions or nanoparticles would also be possible because separation of the particles from the release medium used for sampling is assured when using the EFC. If the results for implants are transferred to nanoparticles, this would probably also be considered with a slower drug release in the EFC compared with compendial methods.

All experiments in this work were performed at room temperature because temperature control of the EFC could not be implemented thus far. This detail is to be seen as a disadvantage compared with the compendial methods, in which the temperature can be easily adjusted. Especially in the dissolution of implants and the disintegration process of polymers, temperature plays a major role. This aspect must be considered in future developments in any case.

Because mainly young animals are used in preclinical in vivo studies, the gel used in this work should take this aspect into account [13,29]. However, using a pure gel body as a vitreous substitute here only represents idealized physiological conditions. With aging, the human vitreous body liquefies [25], so that an applied movement might influence the release behavior because of the addition of convective processes [9]. Because the incidence of many posterior eye diseases (diabetic retinopathy, age-related macular degeneration, and macular edema) increases with age, simulation of a liquefied vitreous should be included in further experiments. A simulation of a vitrectomized vitreous body would also be possible with the EFC. Stein et al. showed that the addition of silicone oil to an in vitro vitreous substitute has an influence on distribution processes [30]. Because of the outer agarose sheath, the inner gel core can be easily adapted to these different conditions.

In conclusion, the EFC, a gel-based flow-through cell, was developed to simulate the human vitreous body in this work. With this, a continuous dissolution study of intravitreal implants is possible without the need for multiple experiments with different endpoints or manual transfer of the implants. In the future, temperature control of the system needs to be considered to take the influence of body temperature into account. Further steps should include the testing of commercially available dosage forms using this setup. However, model evaluation based on the comparison of in vitro and in vivo data is complicated for intravitreal products because in vivo data regarding release into the vitreous are not available owing to the local release of small amounts of drug over time and the inaccessibility of human vitreous for sampling. Nevertheless, the model is a first approach to investigate intravitreal dosage forms under more biorelevant conditions in preclinical development.

## 5. Conclusions

Models of more biorelevant in vitro release in preclinical development can better predict the dissolution behavior of dosage forms in vivo. Official compendial drug dissolution methods generally represent aqueous systems that often inadequately reflect physiological conditions. The EFC developed here as a modified flow-through cell represents a first approach to study intravitreal dosage forms, in which a gel simulates the human vitreous body. The setup was successfully printed with a 3D printer using stereolithography. It was shown that drug release from FS- or TA-containing HPMC implants was significantly slower when using a gel system compared with two standard compendial methods (USP apparatus 4 and 7). The EFC represents a gel-based release system that allows continuous dissolution testing of intravitreal dosage forms. Further studies, specifically on long-term suitability, need to be conducted to further evaluate the system.

## Figures and Tables

**Table 1:** Composition of the modified polyacrylamide gel by Loch et al.

Compound	Content (%)
phosphate buffered saline pH 7.4	92.21
rotiphoresis gel 30	6.69
ammonium peroxodisulfate	1
tetramethylethylendiamine	0.1

**Table 2:** Composition of the powder mixtures for fluorescein sodium and triamcinolone acetonide implants produced via hot-melt extrusion.

Compound	Batch 1 Content (%)	Batch 2 Content (%)
fluorescein sodium	40	-
triamcinolone acetonide	-	20
silicone dioxide	0.5	0.5
polyethylene glycol 6000	10	10
hydroxypropyl methylcellulose	49.5	69.5

**Table 3:** Movement pattern of the applied schematic eye movement.

Movement Pattern (*x*-Axis)		Angle (°)	Angular Velocity (°/s)
**Mode 1**	Slow pursuit movement	35	41
**Mode 2**	Fast pursuit movement	33	83
**Mode 3**	Saccadic movement	42	165
**Mode 4**	Pursuit movement with distinct amplitudes	90	330
**Movement pattern *y*-axis**		20	60
**24 h day rhythm** **Minutes (mode)**		290 min (mode 1), 5 min (mode 3) 280 min (mode 4), 5 min (mode 3) 260 min (mode 2), 5 min (mode 3) 300 min (mode 1), 5 min (mode 3) 285 min (mode 4), 5 min (mode 3)	

## Data Availability

The data presented in this study are available on request from the corresponding author.
